# Supratentorial ependymoma in childhood: more than just RELA or YAP

**DOI:** 10.1007/s00401-020-02260-5

**Published:** 2021-01-22

**Authors:** Valentina Zschernack, Stephanie T. Jünger, Martin Mynarek, Stefan Rutkowski, Maria Luisa Garre, Martin Ebinger, Marie Neu, Jörg Faber, Bernhard Erdlenbruch, Alexander Claviez, Stefan Bielack, Triantafyllia Brozou, Michael C. Frühwald, Evelyn Dörner, Verena Dreschmann, Annika Stock, Laszlo Solymosi, Jürgen Hench, Stephan Frank, Christian Vokuhl, Andreas Waha, Felipe Andreiuolo, Torsten Pietsch

**Affiliations:** 1 Department of Neuropathology, DGNN Brain Tumor Reference Center, University of Bonn Medical Center, Venusberg-Campus 1, 53127 Bonn, Germany; 2 Department of Pediatric Oncology and Hematology, University Medical Center Hamburg-Eppendorf, Hamburg, Germany; 3 Neuro-Oncology Unit, IRCCS Istituto Giannina Gaslini, Genoa, Italy; 4 Department of Pediatric Hematology and Oncology, University Hospital Tübingen, Tübingen, Germany; 5 Department of Pediatric Hematology/Oncology, Center for Pediatric and Adolescent Medicine, University Medical Center of the Johannes Gutenberg-University Mainz, Mainz, Germany; 6 Johannes Wesling Klinikum Minden, University Hospital for Children and Adolescents, Ruhr University Hospital, Bochum, Germany; 7 Department of Pediatric and Adolescent Medicine, Pediatric Hematology and Oncology, University Hospital Schleswig Holstein, Kiel, Germany; 8 Department of Pediatric Oncology, Hematology and Immunology, Center for Pediatric, Adolescent and Women’s Medicine, Stuttgart Cancer Center, Klinikum Stuttgart-Olgahospital, Stuttgart, Germany; 9 Department of Pediatric Oncology, Hematology and Clinical Immunology, University Children’s Hospital Medical Faculty, Heinrich-Heine-University, Düsseldorf, Germany; 10 University Medical Center Augsburg, Swabian Children’s Cancer Center, Augsburg, Germany; 11 Department of Neuroradiology, University Hospital Würzburg, Würzburg, Germany; 12 Division of Neuropathology, Department of Medical Genetics and Pathology, University Hospital Basel, Basel, Switzerland; 13 Pediatric Pathology, Department of Pathology, University of Bonn Medical Center, Bonn, Germany; 14 Present Address: Department of Neurosurgery, University of Cologne Medical Center, Cologne, Germany; 15 Present Address: Instituto Estadual Do Cérebro Paulo Niemeyer and the IDOR Institute, Rio de Janeiro, Brazil

**Keywords:** Ependymoma, Pediatric, Brain tumor, Childhood, Supratentorial, C11orf95 fusion, Methylation profiling, Tanycytic

## Abstract

Two distinct genetically defined entities of ependymoma arising in the supratentorial compartment are characterized by the presence of either a *C11orf95-RELA* or a *YAP-MAMLD1* fusion, respectively. There is growing evidence that supratentorial ependymomas without these genetic features exist. In this study, we report on 18 pediatric non-*RELA/*non-*YAP* supratentorial ependymomas that were systematically characterized by means of their histology, immunophenotype, genetics, and epigenomics. Comprehensive molecular analyses included high-resolution copy number analysis, methylation profiling, analysis of fusion transcripts by Nanostring technology, and RNA sequencing. Based upon histological and immunohistochemical features two main patterns were identified—*RELA*-like (*n* = 9) and tanycytic ependymomas (*n* = 6). In the *RELA*-like group histologically assigned to WHO grade III and resembling *RELA*-fused ependymomas, tumors lacked nuclear expression of p65-RelA as a surrogate marker for a pathological activation of the NF-κB pathway. Three tumors showed alternative *C11orf95* fusions to *MAML2* or *NCOA1*. A methylation-based brain tumor classifier assigned two *RELA*-like tumors to the methylation class “EP, *RELA*-fusion”; the others demonstrated no significant similarity score. Of the tanycytic group, 5/6 tumors were assigned a WHO grade II. No gene fusions were detected. Methylation profiling did not show any association with an established methylation class. We additionally identified two astroblastoma-like tumors that both presented with chromothripsis of chromosome 22 but lacked *MN1* breaks according to FISH analysis. They revealed novel fusion events involving genes in chromosome 22. One further tumor with polyploid cytogenetics was interpreted as PFB ependymoma by the brain tumor methylation classifier but had no relation to the posterior fossa. Clinical follow-up was available for 16/18 patients. Patients with tanycytic and astroblastoma-like tumors had no relapse, while 2 patients with *RELA*-like ependymomas died. Our data indicate that in addition to ependymomas discovered so far, at least two more supratentorial ependymoma types (*RELA*-like and tanycytic) exist.

**Supplementary Information:**

The online version contains supplementary material available at 10.1007/s00401-020-02260-5.

## Introduction

Ependymomas represent the second most common malignant intracranial neoplasms in children and young adults [15]. In childhood, ependymomas occur across all compartments of the central nervous system (CNS); the most frequent location is the posterior fossa followed by supratentorial and spinal sites [13]. Based on histological resemblance, ependymomas across different sites had been considered as one entity in the past. Various underlying genetic changes in ependymomas were incorporated in the 2016 WHO-classification of brain tumors [12]. Based on publications by Parker, Pietsch, and Pajtler the supratentorial ependymoma with *RELA* fusion was introduced as a distinct entity [17–19]. Seven different *RELA*-fusion variants have been identified [18]. These tumors represent approximately 70% of supratentorial ependymomas of childhood [19]. Besides ependymomas with *RELA* fusion, tumors with *YAP1-MAMLD1* fusion were described as a second entity emerging in the supratentorial compartment of pediatric patients [1, 17, 18]. In contrast to *RELA*-fused tumors associated with adverse outcomes [12, 17], *YAP1-MAMLD1* fused ependymomas show an excellent prognosis [1, 17].

According to the methylation-based classification by Pajtler et al., apart from ependymoma with *RELA* and *YAP* fusions, only subependymomas are to be found in the supratentorial compartment [17]. Opposed to this model, recent publications described supratentorial ependymomas lacking *RELA* or *YAP1* fusions [5, 16].

In this study, we identified a cohort of non-*RELA-*non-*YAP* (NRNY) ependymomas and systematically analyzed their clinical, histological, genetic, and epigenomic features.

## Materials and methods

### Patients and tumor material

Between 2003 and 2017 eighteen pediatric NRNY ependymomas with supratentorial location were reviewed at the Brain Tumor Reference Center of the German Society of Neuropathology and Neuroanatomy (DGNN) at the Institute of Neuropathology, University of Bonn Medical Center, Germany. Formalin-fixed, paraffin-embedded (FFPE) material was available from all patients. All examinations were carried out on the basis of and according to the legal requirements of the revised Declaration of Helsinki of the World Medical Association in 1983. Eight patients had also participated in the HIT2000 Ependymoma trial.

### Neuropathological evaluation

Central neuropathological evaluation was performed at the time of diagnosis at the DGNN Brain Tumor Reference Center. The tumors were re-evaluated and classified according to the WHO classification of tumors of the CNS by at least two experienced neuropathologists (TP, FA) after evaluation of HE and immunohistochemically stained slides (GFAP, EMA, p65-RelA, Olig2, MAP2, phosphohistone-H3, p-16, Ki-67) [12]. In this study, specific cytological and histological features were examined in all cases. Immunohistochemical staining—performed on an automated immunostaining system (BenchMark XT, Ventana-Roche, Mannheim, Germany)—included glial fibrillary acidic protein (GFAP; rabbit polyclonal, Agilent/Dako, Glostrup, Denmark), microtubule-associated protein 2 [Map2; mouse monoclonal (HM-2), Sigma, St. Louis, MO, USA], epithelial membrane antigen [mouse monoclonal (E-29), Agilent/Dako, Glostrup, Denmark], Olig2 (goat polyclonal, R&D Systems, Abingdon, UK), Ki-67 (MAb MIB-1; Dako, Glostrup, Denmark), phosphohistone-H3 (Biocare, Concord, USA), p16 protein (MAb E6H4, Ventana-Roche, Darmstadt, Germany), and p65-RelA (rabbit antibody D14E12, Cell Signaling, Danvers, USA) [8, 9].

### DNA extraction and genome-wide copy number analysis

DNA was extracted from FFPE material using the QIAmp DNA FFPE Tissue Kit (Qiagen, Hilden, Germany). Molecular inversion probe analysis (MIP; Oncoscan, ThermoFisher, Waltham, MA, USA) and/or human SNP-6 array (ThermoFisher) and data-mining utilizing Nexus Copy Number 7·0 Discovery Edition software (BioDiscovery, El Segundo, CA, USA) was performed as previously described [10].

### RNA extraction and analysis for RELA and YAP fusion transcripts

RNA was isolated from FFPE tumor probes using the AllPrep DNA/RNA FFPE kit (Qiagen, Venlo, The Netherlands). The tumors were further tested for the presence of *C11orf95-RELA* and *YAP1-MAMLD1* fusions by RT-PCR, as published previously [1, 4, 9, 19]. In addition, Nanostring analysis was performed. The Nanostring fusion panel covers 88 recurrent fusions present in brain tumors, including the four most frequent *C11orf95-RELA* fusions (*C11orf95-RELA*, exon 2–exon 2; exon 2–exon 3; exon 3–exon 2; exon 3–exon 3) as well as a *YAP1-MAMLD1* fusion (exon 5–exon 3) and a *YAP1-FAM118B* (exon 7–exon 3) fusion.

### mRNA NGS fusion panel

Targeted RNA sequencing was performed using the TruSight RNA Fusion Panel (Illumina, San Diego, CA, USA) according to the manufacturer’s protocol. In brief, 100 ng RNA was used for library preparation. Libraries were then hybridized to biotin-labeled probes specific for targeted RNA regions. By adding streptavidin beads targets are captured and then magnetically enriched. Cluster generation and sequencing were then performed on the MiSeq System (Illumina). Subsequently, bcl files were processed into fastq files and analysis was performed on the Illumina platform BaseSpace using the RNA-Seq Alignment v2.0.1.

### mRNA expression of lineage-related genes

Expression of specific gene transcripts was analyzed using a custom-made panel with the nCounter Elements technology (Nanostring Technologies, Seattle, WA, USA) following the manufacturer’s protocol. Data analysis was performed with the nSolver analysis software (Nanostring Technologies). The assay includes probes for the quantification of mRNA species characteristically expressed in brain tumors including *MUC1* (EMA), *OLIG2, RELA, TNC*, and *BEND2* (associated to *MN1*-related tumors).

### DNA methylation analysis

250–500 ng of tumor DNA was converted by bisulfite treatment and analyzed on the Infinium Human Methylation EPIC (850 k) or 450 (450 k) BeadChip (Illumina, San Diego, CA, USA) according to the manufacturer’s protocol. Methylation data obtained from the array was analyzed with the Heidelberg methylation brain tumor classifier, version v11b4 (www.molecularneuropathology.org), which is built upon supervised machine learning [3]. In parallel, the methylation array data were compared to > 15.500 methylome datasets across the entire spectrum of human neoplasias and control tissues by unsupervised machine learning in the form of dimension reduction with UMAP plots (www.epidip.org, v. 4.2 GPU, 25,000 top differentially methylated CpGs) [20].

### *Fluorescence *in situ* hybridization*

Break-apart probe fluorescence in situ hybridization (FISH) of the *MN1* locus was performed in two cases on paraffin sections using the *MN1* break-apart FISH probe (Cytotest, Rockville, MD, USA). When analyzed cells showed a split of at least one set of red and green signals or an isolated (red or green) signal, FISH was considered positive.

### Radiologic evaluation

Initial MR-imaging studies of a subset of ependymomas were retrieved from the database of the National Reference Center for Neuroradiology (Department for Neuroradiology; Würzburg University Hospital, Germany) and reviewed by an experienced neuroradiologist (AS). Features evaluated were supratentorial localization (intracerebral vs. intraventricular); homogeneity/heterogeneity on T2-weighted images; perifocal tumor edema (yes/no); restriction on diffusion-weighted images (yes/no); intratumoral blood degradation products on T2- or T2*-weighted images (yes/no); cystic/necrotic components (< 50% vs. > 50% of the tumor volume). Contrast agent uptake of the tumor was compared to strong enhancing tissue like nasal mucosa and rated as mild, moderate or strong.

### Statistics and survival analysis

Progression-free and overall survival (PFS/OS) was defined as the time from surgery to the first event (progression/relapse/death) or the date of the last follow-up. We constructed Kaplan–Meier curves and compared patient groups with log-rank tests. A two-sample *t *test allowing unequal variance (Welch’s *t *test) was performed to detect significant age differences between two groups; a *p *value < 0.05 was considered significant.

## Results

### Patient demographics and histological findings

Of all supratentorial ependymomas in children diagnosed between 2003 and 2017 at the DGNN brain tumor reference center, approximately 15% harbored neither a *RELA* nor a *YAP* fusion.

**Fig. 1:**
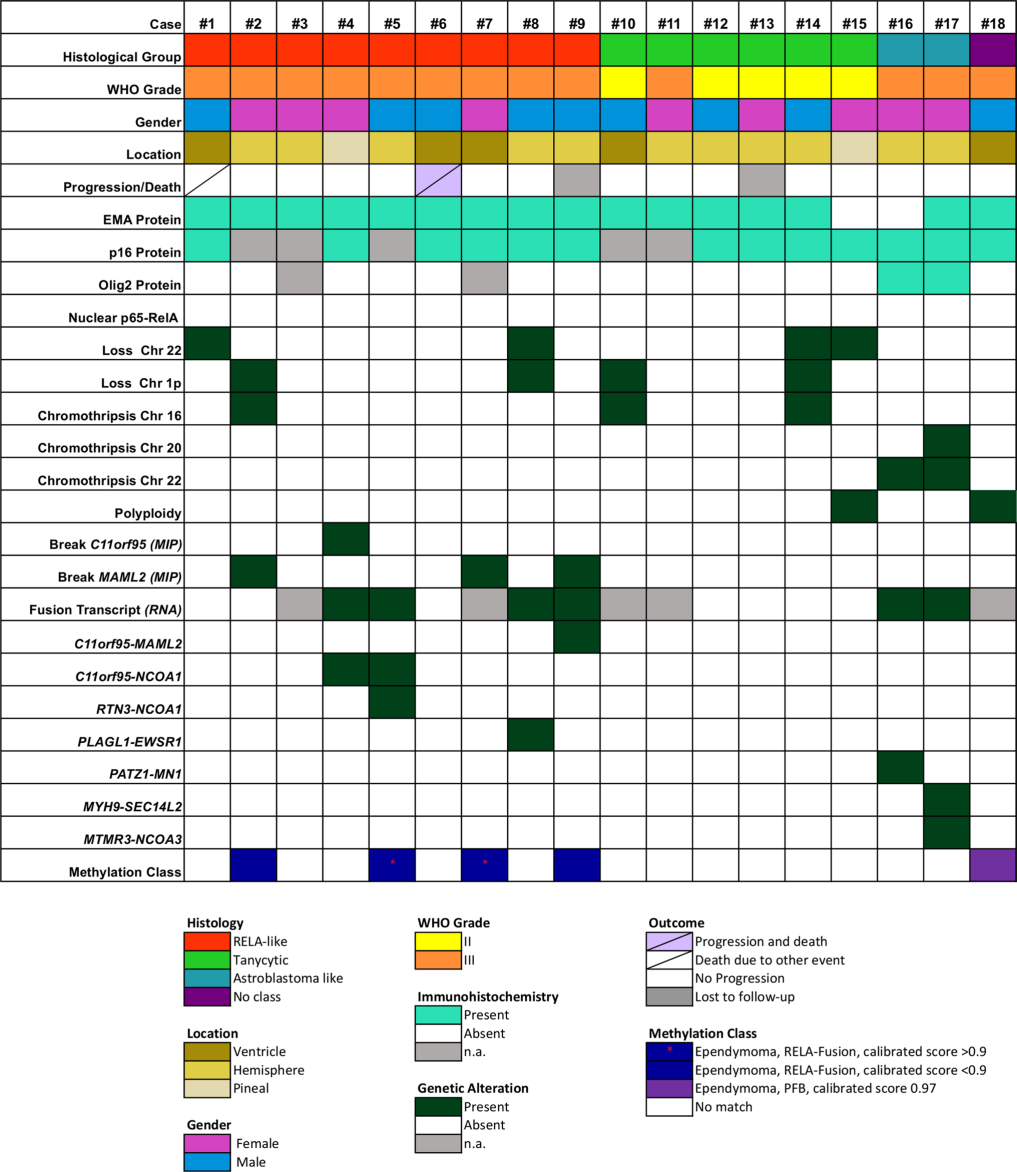
Clinical, histological, and genetic features of NRNY tumors. Histological class, WHO grade, gender, localization, and immunohistochemical features of all 18 tumors are shown in the top columns. The most frequent copy number alterations [detected with molecular inversion probe assay (MIP)] and gene fusion events (RNA-NGS) are shown in the middle panel. In the bottom, results from the methylation analysis are outlined

All 18 tumors of this cohort were localized in the supratentorial compartment. The median age at the time of diagnosis was 8.3 years ranging from 0.5–17.8 years. Gender distribution was equal with nine female and nine male patients; both patients with astroblastoma-like tumors were female; for detailed information refer to Table 1 and Fig. 1. One female patient was diagnosed with neurofibromatosis type II and developed three additional meningiomas as well as a neurinoma (case 15).

**Table 1:** Patient characteristics, survival data, and treatment information

No.	Gender	Location	WHO grade	Age at operation (years)	EFS (years)	Event	OS (years)	Outcome	Subgroup	Postoperative treatment
1	M	3rd ventricle	III	12.80	7.43	Second tumor	7.62	Dead	*RELA*-like	HIT-2000
2	F	Hemispheric	III	4.24	11.68	Censored	11.68	Alive	*RELA*-like	HIT-2000
3	F	Hemispheric	III	1.90	7.68	Censored	7.68	Alive	*RELA*-like	HIT-2000
4	F	Pineal region	III	7.18	8.53	Censored	8.53	Alive	*RELA*-like	HIT-2000
5	M	Hemispheric	III	17.30	7.23	Censored	7.23	Alive	*RELA*-like	HIT-2000/E-HIT 2004
6	M	Lateral ventricle	III	0.52	4.90	Relapse	5.60	Dead	*RELA*-like	VEC-Chemotherapy; UKCCSG/SIOP
7	F	Lateral ventricle	III	4.20	5.49	Censored	5.49	Alive	*RELA*-like	HIT-2000
8	M	Hemispheric	III	6.55	7.48	Censored	7.48	Alive	*RELA*-like	HIT-2000
9	M	Hemispheric	III	2.32	n.a.	n.a.	n.a.	n.a.	*RELA*-like	n.a.
10	M	3rd ventricle	II	15.00	10.40	Censored	10.40	Alive	Tanycytic	HIT-2000
11	F	Hemispheric	III	13.64	5.58	Censored	5.58	Alive	Tanycytic	I-HIT-MED
12	M	Hemispheric	II	14.90	3.38	Censored	3.38	Alive	Tanycytic	I-HIT-MED
13	F	Hemispheric	II	6.90	n.a.	n.a.	n.a.	n.a.	Tanycytic	n.a.
14	M	Hemispheric	II	17.80	2.80	Censored	2.80	Alive	Tanycytic	Irradiation only
15^a^	F	Pineal region	II	10.30	6.90	Censored	6.90	Alive	Tanycytic	Irradiation only
16	F	Hemispheric	III	13.50	6.78	Censored	6.78	Alive	Astroblastoma-like	HIT-2000
17	F	Hemispheric	III	5.25	8.61	Censored	8.61	Alive	Astroblastoma-like	HIT-2000
18^b^	M	3rd ventricle	III	9.38	1.70	Censored	1.70	Alive	Other	I-HIT-MED

*NF2* neurofibromatosis type 2; *VEC* vincristine, etoposide, cyclophosphamide; *PFB* posterior fossa ependymoma, group B

^a^NF2 patient

^b^Genetically classified as PFB ependymoma

**Fig. 2:**
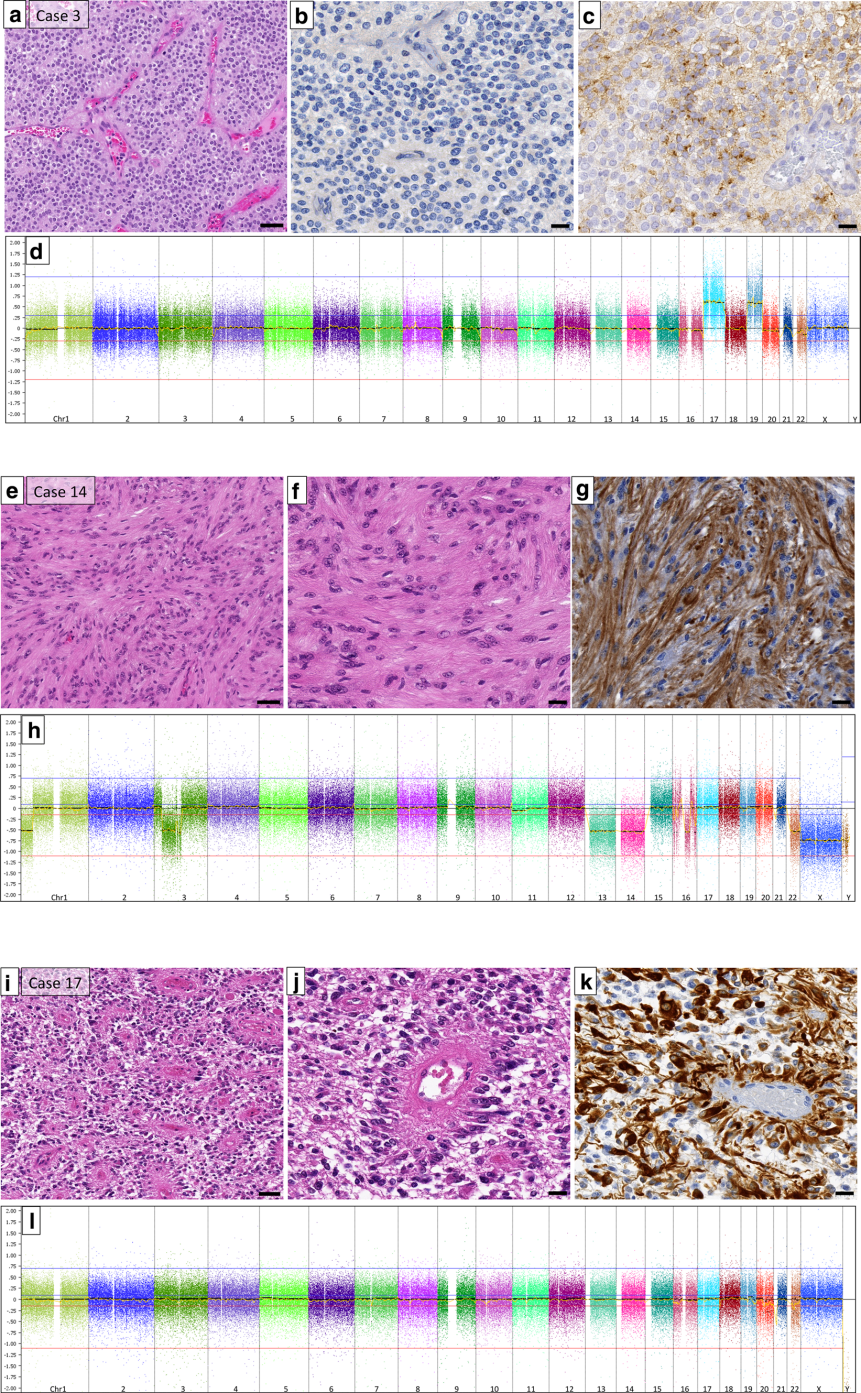
Histological and genetic characteristics of *RELA*-like, tanycytic, and astroblastoma-like tumors. **a** Haematoxylin/eosin (HE) staining of case 3 (*RELA*-like group). **b** RelA immunohistochemistry displaying no nuclear accumulation of the p65-RelA protein. **c** EMA positivity in some tumor cells. **d** genomic copy number profile of case 3 showing gains of chromosomes 17 and 19 but an otherwise balanced genome. **e**, **f** HE staining of a tanycytic ependymoma (case 14) with typical elongated cell processes highlighted in the GFAP immunohistochemistry (**g**). **h** copy number profile of this case showing copy number losses of 1p and 3p as well as chromosomes 13, 14, and 22, and chromothripsis of chromosome 16. **i**, **j** HE of case 17 illustrating the astroblastoma-like appearance, strongly highlighted in the GFAP immunohistochemistry (**k**). **l** copy number profile of case 17 showing structural alterations of chromosomes 20 and 22 (chromothripsis) (*scale bars* in **a**, **e**, **i**: 50 µm; *scale bars* in **b**, **c**, **f**, **g**, **j**, **k**: 20 µm)

According to their histological features, three different characteristic histological patterns were identified: *RELA*-like (*n* = 9), tanycytic (*n* = 6), and astroblastoma-like (*n* = 2) histology (Figs. 1, 2). One ependymoma did not show a specific histological pattern. Patients in the *RELA*-like group were significantly younger at the time of diagnosis compared to those in the tanycytic group (mean age 6.33 years and 13.09 years, respectively, Welch’s *t *test, two-sided *p* = 0.015). Thirteen patients were treated according to HIT-ependymoma standard protocols (ClinicalTrials.gov NCT00303810); two patients received radiotherapy only and one patient received chemotherapy and radiotherapy, and was later treated according to the UKCCSG/SIOP protocol [7].

The ependymomas with *RELA*-like histology often presented with densely packed small cells with round nuclei, frequent clear cell morphology and branching capillaries reminiscent of the typical morphology *of RELA* fusion-positive ependymomas. Microcystic components were observed in cases 2 and 8. Case 4 displayed foci of calcification and single giant tumor cells. Areas of necrosis were observed in cases 3 and 6. Cases 6 and 7 also demonstrated notable endothelial proliferation. In five tumors, the samples contained areas from the border zone of the tumor to the adjacent brain. Only two of the *RELA*-like tumors (cases 4 and 8) displayed small clusters of cells infiltrating the brain tissue, the other three showed a clear demarcation (cases 5, 14 and 15). Thirteen samples only contained solid tumor areas; there was no indication of a diffusely infiltrative growth pattern. Preexisting neurons or axonal structures could not be identified within the tumors.

Dot-like EMA expression was found in all *RELA*-like tumors. All tumors were anaplastic (WHO grade III). None of the tumors showed nuclear p65-RelA protein accumulation (Fig. 1). p16 protein was retained in all cases that were examined by immunohistochemistry. In summary, there was no obvious histological difference of these tumors compared to *RELA*-fused ependymomas with the exception of the absence of nuclear p65-RelA accumulation.

Ependymomas with tanycytic histology displayed round to oval, mostly isomorphic cell nuclei and elongated cell processes, particularly highlighted by GFAP immunostaining. Some perivascular pseudorosettes were observed but did not represent a major histological feature. One tanycytic tumor (case 10) presented with fiber-rich areas and protein droplets, and case 11 exhibited a prominent microcystic component (Fig. 2). Five out of 6 tanycytic tumors were assigned a WHO grade II according to the 2016 WHO classification of brain tumors [12]. All five cases tested showed a dot-like EMA expression, p16 protein expression, and a lack of nuclear p65-RelA accumulation.

The two tumors with astroblastoma-like histology (Fig. 2) presented with perivascular architectures resembling astroblastic pseudorosettes with long, stamp-like processes directed towards the vessel walls. One case (case 16) showed foci of hyalinized vessels. Both astroblastoma-like tumors were graded as anaplastic and exhibited areas of necrosis. In contrast to the other cases, subpopulations (approximately 20%) of tumor cells expressed Olig2. One case showed an EMA expression (Fig. 1).

The remaining tumor could not be assigned to any of these histological patterns and corresponded to WHO grade III. It exhibited perivascular pseudorosettes, prominent ependymal lining, and dot-like EMA expression (Supplementary Fig. 1, online resource).

### Genomic analysis

High resolution, genome-wide chromosomal copy number analysis by MIP was available for all 18 cases and displayed chromosomal gains and losses, predominantly involving whole chromosomes or chromosomal arms. The most frequent events were loss of chromosome 22 (*n* = 4; 22%) and loss of the short arm of chromosome 1p (*n* = 4; 22%). These occurred in *RELA*-like and tanycytic tumors. Cases 1 and 8 of the *RELA-*like group presented with multiple chromosomal gains and losses (among others, losses of chromosomes 13 and 22; gains of chromosome 21). Chromothripsis of chromosome 11 or focal copy number alterations of the *RELA* locus was not present in any of these cases. In contrast, a chromosomal break in the *MAML2* locus was found in three cases of the *RELA*-like group. Of note, both astroblastoma-like tumors harbored a severe structural alteration (chromothripsis) of chromosome 22 (Figs. 1, 2). The two astroblastoma-like tumors were additionally analyzed by FISH; no split signals were detected with a *MN1* break-apart probe. Three tanycytic tumors showed balanced cytogenetic profiles, one harbored only a loss of the short arm of chromosome 1. Case 14 of the tanycytic group displayed multiple chromosomal losses including the short arm of chromosomes 1 and 3, copy number losses of chromosomes 13, 14, and 22, and chromothripsis of chromosome 16. The remaining tanycytic case 15 and case 18 of the group without histological class showed a polyploid phenotype.

### Analysis of fusion transcripts

By Nanostring technology, we searched for recurrent fusion transcripts including the most frequent fusions of *C11orf95-RELA* and *YAP1-MAMLD1.* None of the examined tumors showed a signal for these fusions. Targeted RNA next-generation sequencing was performed for 13 (7 *RELA*-like, 4 tanycytic, 2 astroblastoma-like) tumors (Fig. 1). Two *RELA*-like ependymomas harbored a gene fusion involving *C11orf95* with breakpoints in exon 5 and the *NCOA1* gene, breakpoints in exon 14 (case 4) or exon 15 (case 5). An additional *RTN3* (exon 5) - *NCOA1* (exon 15) fusion transcript was found in case 5. Sequencing data revealed a *C11orf95* (exon 5)-*MAML2* (exon 2) fusion in case 9 (Fig. 1). A *PLAGL1* (exon 4) - *ESWR1* (exon 8) gene fusion was detected in case 8. Cases 1, 2, and 6 (RELA-like) as well as cases 12, 13, 14, and 15 (tanycytic) did not show any relevant fusion transcripts in RNA sequencing. Both astroblastoma-like tumors displayed novel gene fusions with case 16 harboring a unique *PATZ1* (exon1)-*MN1* (exon1) gene fusion and case 17 carrying *MYH9* (exon 3)-*SEC14L2* (exon 2) and *MTMR3* (exon 2)-*NCOA3* (exon 10) fusion transcripts.

### mRNA expression of lineage markers

Furthermore, both astroblastoma-like tumors did not show *BEND2* expression characteristic of HGNET-MN1. *OLIG2* mRNA was absent from cases of the *RELA*-like and tanycytic ependymomas but expressed in the two astroblastoma-like tumors.

### Methylation-based classification

For two tumors of the *RELA*-like group methylation-based classification (v11b4) yielded a significant similarity with the methylation class ‘ependymoma, *RELA*-fusion’ (calibrated-score > 0.9); one additional case showed a calibrated-score of 0.89 (case 2) and one further *RELA*-like case showed a low, non-significant, calibrated score of 0.62 for this methylation group. Case 18, located solely in the third ventricle with no relation to the posterior fossa, scored high (0.97) for the methylation class ‘ependymoma, posterior fossa group B’. All tanycytic ependymomas, the two astroblastoma-like tumors as well as all remaining *RELA*-like cases scored ‘no match’. In dimension reduction (www.epidip.org; v. 2.4), 7/8 *RELA*-like ependymomas clustered with *RELA*-fused ependymomas. One tumor (case 4) did not cluster with them, but showed typical histology with some clear cell-like areas and branching capillaries. This case also carried an alternative *C11orf95* fusion. Five of six tanycytic ependymomas did not group with any of the molecularly distinct ependymoma entities but showed a certain similarity to other low-grade gliomas (Supplementary Fig. 2, online resource). Three of the tanycytic cases showing a stable genome clustered together in the UMAP plot.

### Imaging characteristics

Initial MRIs of *RELA*-like ependymoma were available for six patients but neuroradiological reevaluation could only be performed in four cases only due to an incomplete imaging protocols and/or defective storage medium. Predominant cystic/necrotic morphology, heterogeneous T2-weighted signal-intensity, and perifocal tumor edema were present in 3/4 cases. Localization of these three tumors was the parietal lobe. Solely one tumor showed only one small cyst, no edema, homogeneous T2-weighted signal-intensity and was located in the ventricle. The tumor’s contrast-agent uptake in each case was mainly strong in nearly 100% of the tumor volume. In two cases signs of calcification and/or intratumoral hemorrhage were found, in the other two cases, these features could not be determined. In two patients diffusion-weighted images were available, both tumors showed restricted diffusion.

### Event-free and overall survival

**Fig. 3:**
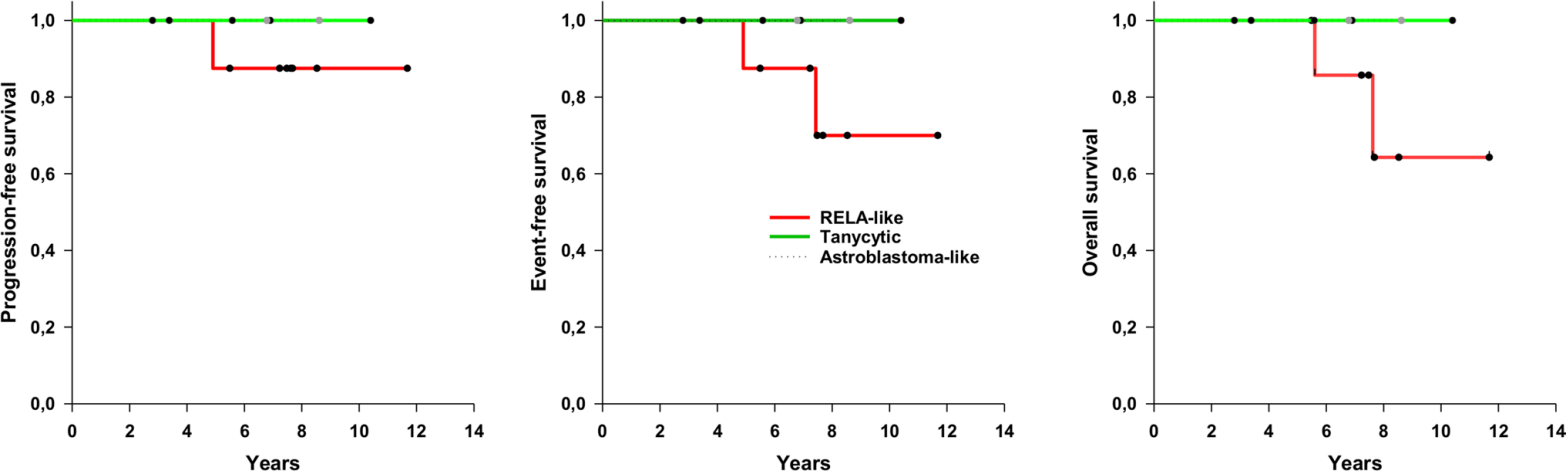
Survival analysis (Kaplan–Meier plots)

The mean follow-up period was 6.73 years (SE 0.79); two patients were lost to follow-up. Of the remaining sixteen patients, at the time of analysis, one had relapsed 4.9 years from diagnosis, and one patient developed a second tumor (astrocytic glioma); these patients, both male, died after 5.6 and 7.6 years, respectively. Five-year EFS, PFS, and OS comprised 75%, 75%, and 81%, respectively. Regarding histological subgroups, in the *RELA*-like group one of eight patients relapsed, one developed a second neoplasm and both died (no survival data available for one patient) while no patient in the tanycytic group experienced relapse nor died (follow-up data missing for one patient) (Table 1). The same applied for the two patients with astroblastoma-like tumors of whom neither one relapsed nor died (Fig. 3).

## Discussion

As the molecular era of brain tumor classification advances, new tumor entities are defined according to genetic alterations. The current concept implies supratentorial ependymomas comprising *C11orf-RELA*-fused or *YAP1-MAMLD1*-fused ependymoma as well as subependymoma [17]. However, growing evidence suggests that supratentorial ependymomas carrying alternative gene fusions exist [5, 16]. To further examine this issue, we studied 18 supratentorial pediatric ependymoma diagnosed between 2003 and 2017 at our institution identified as *C11orf95-RELA* and *YAP1-MAMLD1* fusion-negative. To our knowledge, this study represents the largest cohort of pediatric supratentorial ependymomas lacking *RELA* and *YAP* fusions. A summary of the non-RELA / non-YAP1 supratentorial tumors published so far and the cases of this series is provided in Supplementary Table 1 (online resource).

First, we confirmed the diagnosis by histopathological review including immunohistochemistry for EMA as well as Olig2, with the latter being partly positive only in both astroblastoma-like cases. In all cases, immunohistochemistry for p65-RelA was performed, a potent diagnostic tool to identify *RELA*-positive ependymoma, independent of the underlying fusion transcript [6]. None of the cases showed positive nuclear staining in line with the absence of *C11orf95-RELA* fusion transcripts. *YAP1-MAMLD1* fusions were not found in any case. After confirmation of the absence of these fusions, we identified histological characteristics of the 18 NRNY tumors and were able to assign most tumors to three groups based upon common histological features: the *RELA*-like, the tanycytic, and the astroblastoma-like. Only one case did not fit into any of these groups. None of the tumors displayed features of subependymoma. Thirteen out of 18 (72%) NRNY ependymoma displayed anaplastic features with brisk mitotic activity and graded according to WHO grade III, only five cases qualified for WHO grade II. Interestingly, all five WHO grade II ependymoma presented with a tanycytic phenotype, whereas all *RELA*-like as well as the astroblastoma-like tumors were classified WHO grade III. Referring to survival data—in line with the more favorable grading—tanycytic ependymoma may comprise a favorable subgroup of supratentorial ependymoma.

### RELA-like ependymoma

All nine *RELA*-like ependymomas were classified as WHO grade III. Tumors were localized throughout the supratentorial compartment. They exhibited histological similarities with the *RELA*-fusion positive ependymoma and EMA immunohistochemistry was positive in all cases. With the exception of the lack of nuclear p65-RelA accumulation, there were no differences between *RELA*-like and *RELA*-fused ependymomas regarding histological or immunophenotypical features.

Two cases harbored loss of chromosome 22 and of the short arm of chromosome 1 each; chromothripsis of chromosome 16 was present in two cases, whereas such alteration was not found in chromosome 11, as it would be typical for *RELA*-fusion positive ependymoma. Beyond that, breaks in the *MAML2* and the *C11orf95* locus in three and one cases, respectively, were detected, but no breaks within the *RELA* locus. Such breaks were not found in any of the tumors assigned to the other groups, possibly representing a distinct characteristic feature of *RELA*-like ependymoma.

In addition to the histopathological and genetic analyses, we added methylation-based classification [17]. However, using the (supervised) Heidelberg brain tumor methylation classifier v11b4, only four histologically *RELA*-like tumors were attributed to the methylation class “*RELA*-Fusion EP” (two with a non-diagnostic score of < 0.9). Of these, three showed an alternative *C11orf95* fusion confirmed by RNA-sequencing. It can be hypothesized that *C11orf95* fusion events with alternative partners (*MAML*2 and *NCOA1*) may be associated with a methylation profile similar to cases in which *RELA* is part of the fusion transcript. In fact when we applied unsupervised machine learning in the form of dimension reduction to the methylation data along with a larger spectrum of methylation reference data [20] all but one case of the *RELA*-like ependymomas located within the cluster of *RELA*-fused ependymomas (Supplementary Fig. 2, online resource). This finding may indicate a common cell of origin of *RELA*-fused and *RELA*-like ependymomas.

However, *RELA*-like ependymoma did not show the pathological NF-κB pathway activation characteristic of *RELA*-fused ependymomas and therefore may represent a different biologic entity with distinct clinical behavior.

Zhu et al. recently demonstrated that the C11orf95 portion of the C11orf-RelA fusion protein binds to specific DNA motifs and regulates expression of a set of genes by hijacking the activation domain of RelA [23]. They postulated that C11orf95 may play a driving role in the development of these supratentorial ependymomas because it was found to be fused to other transcription factors such as Yap1 [18] leading to activation of alternative pathways other than NF-κB. Interestingly, other fusion partners of *C11orf95* encode coactivators such as NCOA1, that is known to enhance transcription activation by nuclear hormone receptors [22] or MAML2 that enhances Notch-mediated activation of transcription [11]. These coactivators may be hijacked by C11orf95 and located to other DNA domains leading to activation of an oncogenic transcriptional program. On the other hand, some *RELA*-like tumors carried different alternative fusion transcripts neither harboring *RELA* nor *C11orf95* as well; with a fusion between *RTN3*- and *NCOA1* in case 5 and one *PLAGL1-EWSR1* fusion in case 8. Although we found such alternative fusion transcripts in supratentorial NRNY ependymoma, we did not detect the previously published ones: *FOXO1-STK24* [5], *MAML2-ASCL2,* or *MARK2-ADCY3* [16].

Furthermore, several *RELA*-like tumors were found by Pages et al. and in this series which lacked any detectable fusions (case 23 in the Pages et al. series [16], cases 1, 2, 6, 9 in this series) indicating that C11orf95 may play an important role in some *RELA*-like tumors but not in all (for details, see Supplementary Table 1, online resource).

On MRI, *RELA*-fused ependymomas were described as heterogeneous masses with predominantly cystic or necrotic morphology, showing mainly strong contrast-agent uptake and diffusion restriction (reflecting high cellularity), and in more than half of the cases intratumoral hemorrhages and calcifications were found [14]. The radiological findings of the limited number of *RELA*-like ependymoma in our cohort do not seem to differ substantially compared to the features reported by Nowak et al. [14].

### Tanycytic ependymoma

All but one tumor in this group (83%) were classified as WHO grade II. Four tumors were localized in the hemispheres. Genetically, there were no distinct alterations, only present in tanycytic ependymoma; however, 50% of cases revealed a completely balanced genomic phenotype. No gene fusion transcripts were detected in four tanycytic tumors examined by RNA-NGS. Regarding clinical outcome, all patients of whom follow-up was valid were alive without progression, including the patient suffering from the tumor classified as WHO grade III (Fig. 3). This finding may indicate a favorable clinical behavior, however, the number of cases in this cohort is too small to draw any definitive conclusions. The identification of such cases in other cohorts will allow a better understanding of the clinical behavior of this group of tumors.

Interestingly, none of the tumors could be assigned to a methylation class defined in the Heidelberg classifier (v11b4), indicating that tanycytic ependymoma may not resemble a “pattern” of ependymoma but rather represent one or more biological entity/entities not yet represented in the v11b4 reference tumor set. In a dimension reduction plot, five of six cases did not cluster to any defined class of ependymomas (Supplementary Fig. 2, online resource). These findings suggest that tanycytic ependymomas represent distinct supratentorial ependymomas featuring a characteristic histomorphology and whose methylation signatures differ from other currently recognized ependymoma entities.

### Astroblastoma-like tumors

Both astroblastoma-like cases arose in female patients, were located in the hemispheres and interpreted as WHO grade III. Histological hallmarks of these tumors were astroblastic pseudorosettes; one case even presented with hyalinized blood vessels. Of note, these were the only tumors showing a partial positivity for the glial transcription factor Olig2. Furthermore, both tumors showed chromothripsis of chromosome 22 and could not be assigned to any established methylation class, in particular not to the methylation class HGNET-MN1. Genetic distinction from astroblastoma was further supported by a negative break-apart-*FISH* analysis for *MN1*, and no elevation of *BEND2* [21]. One case of this group harbored a unique *PATZ1-MN1* gene fusion that has been recently described for the first time in a malignant brain tumor [2]. The second astroblastoma-like tumor harbored two novel gene fusions (*MYH9-SEC14L2* and *MTMR3-NCOA3*). Interestingly all but one gene fusion partner in both astroblastoma-like tumors were located on chromosome 22, thus leading to the assumption that these fusions might be due to the observed structural alteration of chromosome 22 (chromothripsis).

Both astroblastoma-like tumors differed from the other NRNY supratentorial ependymomas. In contrast to these, the astroblastoma-like tumors expressed *OLIG2* transcripts and Olig2 protein in a fraction of cells. Whether these tumors should be classified as an ependymoma subgroup or may represent a novel entity remains to be defined.

### Supratentorial ependymoma without annotation to a distinct histological class

Even though the supratentorial location was radiologically confirmed, one case without annotation to a distinct histological class was classified as “PFB” ependymoma by methylation profiling. In concordance with the methylation class, MIP revealed a polyploid chromosomal genotype, suggesting that rare supratentorial variants of PFB due to aberrant cell migration might exist.

In conclusion, we identified a cohort of 18 pediatric supratentorial NRNY ependymomas and further allocated these tumors to three groups according to their predominant histological features. They seem to represent different biological entities. In our cohort they corresponded to approximately 15% of pediatric supratentorial ependymomas, similar to reports by other groups [5, 16]. It is surprising that these tumors had not been identified in the large cohort of more than one-hundred supratentorial ependymomas described by Pajtler et al. [17]. Currently, the exclusive use of methylation-based classification seems insufficient to distinguish these NRNY ependymomas. We suggest an integrated histopathological and genetic diagnostic work-up of supratentorial ependymomas to precisely identify these tumors to enable their further characterization, eventually leading to adequate risk-stratification in the future.

## Supplementary Information

Below is the link to the electronic supplementary material.

Supplementary file1 (PDF 2578 KB)
