# Supplementary material for: Supratentorial ependymoma in childhood: more than just RELA or YAP
Source: Acta Neuropathol. 2021 Jan 22;141(3):455–66. doi: 10.1007/s00401-020-02260-5 (PMC7882569; doi:10.1007/s00401-020-02260-5)
Supplement: Supplementary file 1 — Supplementary file1 (PDF 2578 KB) [file 401_2020_2260_MOESM1_ESM.pdf]

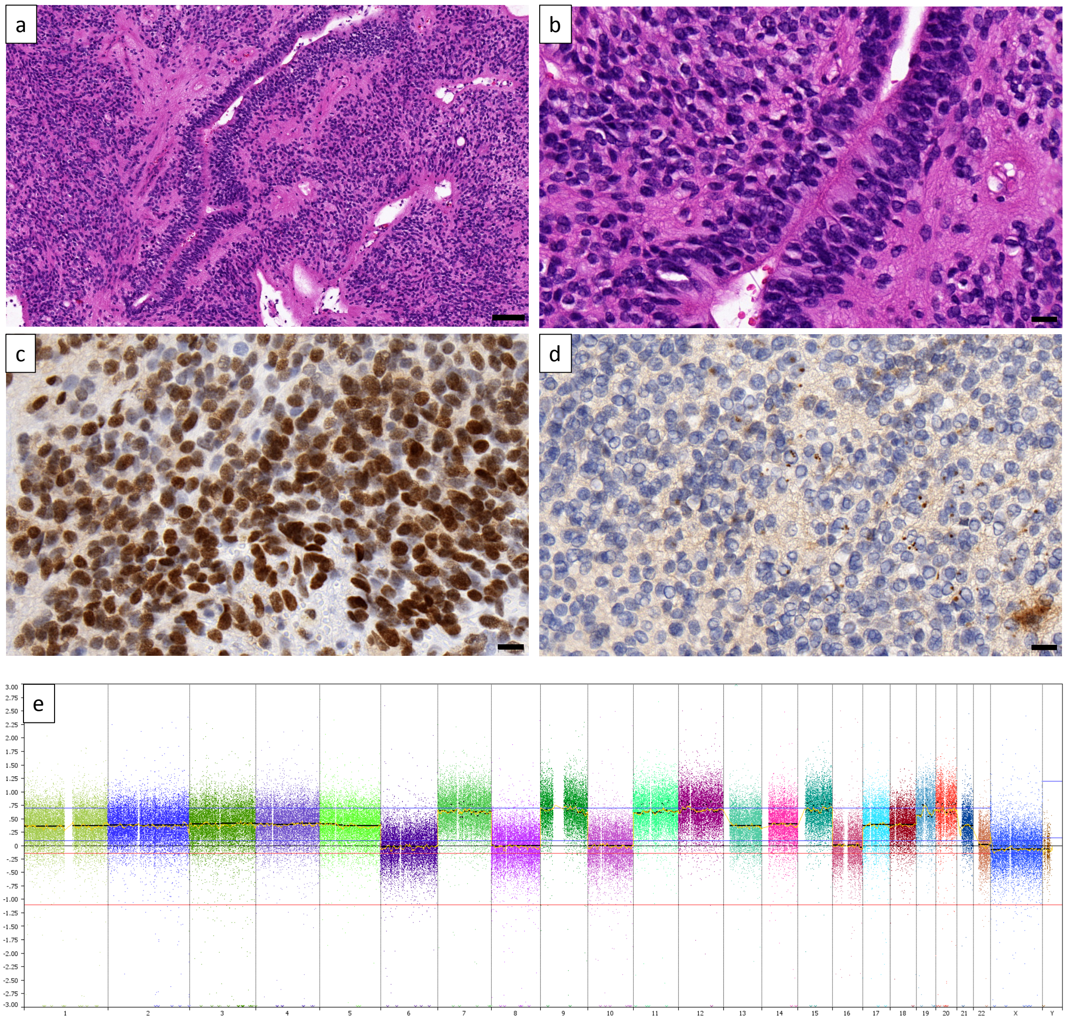

Supplementary Figure 1.

Histological and genetic characteristics of case 18.

a, b HE stains of case 18 (PFB) showing a densely cellular tumor with round to oval cell nuclei and eosinophilic cytoplasms. **c** retained K27-trimethylation of histone 3 lysine (H3K27me^3). **d** dot-like protein expression of epithelial membrane antigen. **e** copy number profile of case 18 highlighting the typical polyploid phenotype with multiple copy number gains. (*scale bar* in **a**: 50 µm, *scale bars* in **b, c, d**: 20 µm)

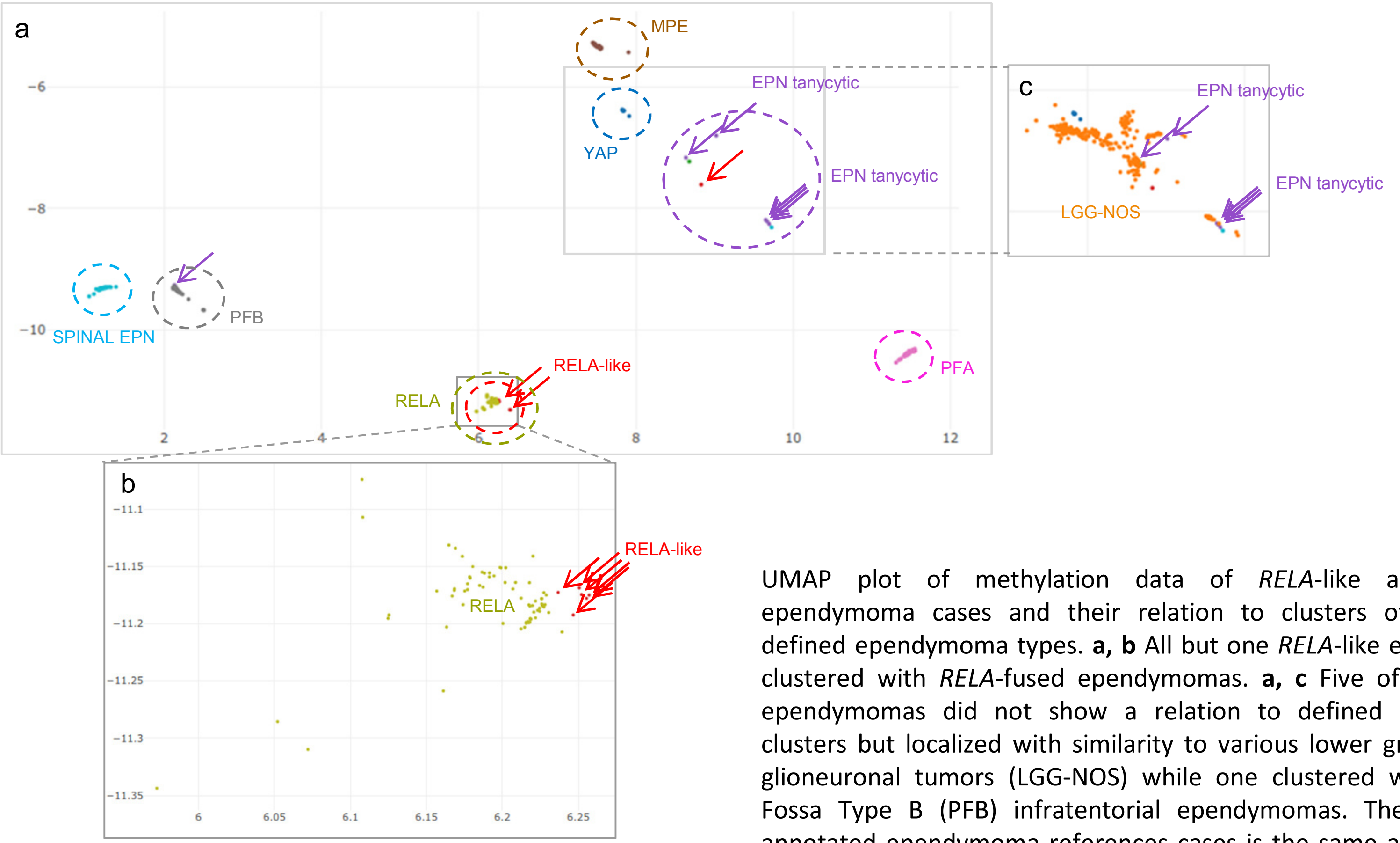

Supplementary Figure 2

UMAP plot of methylation data of *RELA*-like and tanycytic ependymoma cases and their relation to clusters of molecularly defined ependymoma types. **a, b** All but one *RELA*-like ependymomas clustered with *RELA*-fused ependymomas. **a, c** Five of six tanycytic ependymomas did not show a relation to defined ependymoma clusters but localized with similarity to various lower grade glial and glioneuronal tumors (LGG-NOS) while one clustered with Posterior Fossa Type B (PFB) infratentorial ependymomas. The majority of annotated ependymoma references cases is the same as in the brain tumor methylation classifier reference set v11b2. **b** shows the UMAP plot region of *RELA/RELA*-like tumors at higher magnification; **c** shows the location of supratentorial tanycytic ependymomas in relation to low-grade gliomas (masked in **a**). RELA, *RELA*-fused ependymoma; RELA-like, supratentorial *RELA*-like ependymomas, EPN-tanycytic, supratentorial tanycytic ependymoma; MPE, myxopapillary ependymoma; PFA, posterior fossa type A ependymoma; PFB, posterior fossa type B ependymoma; SPINAL EPN, spinal ependymoma; YAP, *YAP*-fused ependymoma; LGG-NOS, low grade glioma, not otherwise specified.

	Age at dx (years)	Gender	Location	Histology	WHO grade	Nuclear p65-RelA	Methylation-based classification	Gene Fusions	Integrated Diagnosis	Treatment	EFS (years)	Event	OS (years)	OS status	Study reference	Study Case No.
1	1	n.a.	thalamic -intraventricular	classic	III	negative	EPN, RELA	no fusion	non-RELA/non-YAP EP	Chemotherapy	n.a.	n.a.	n.a.	n.a.	Pages et al [16]	23
2	1	n.a.	tectal	classic	III	negative	undetermined	n.a.	non-RELA/non-YAP EP	Irradation and chemotherapy	n.a.	n.a.	n.a.	n.a.	Pages et al [16]	24
3	8	n.a.	frontal	classic	III	negative	undetermined	*MAML2-ASCL2*	non-RELA/non-YAP EP	Chemotherapy	n.a.	n.a.	n.a.	n.a.	Pages et al [16]	25
4	10	n.a.	3rd ventricle	classic	III	negative	undetermined	*MARK2-ADCY3*	non-RELA/non-YAP EP	Irradiation	n.a.	n.a.	n.a.	n.a.	Pages et al [16]	34
5	11	n.a.	parieto-temporal	papillary	III	negative	undetermined	n.a.	non-RELA/non-YAP EP	Irradation and chemotherapy	n.a.	n.a.	n.a.	n.a.	Pages et al [16]	35
6	2	n.a.	frontal	classic	III	n.a.	n.a.	n.a.	non-RELA/non-YAP EP	None	n.a.	n.a.	n.a.	n.a.	Pages et al [16]	36
7	56	m	temporal	n.a.	III	n.a.	no match (low score for glioblastoma, IDH wildtype, subclass RTK II, 0.51)	n.a.	ST EP	n.a.	n.a.	n.a.	n.a.	n.a.	Fukuoka et al [5]	EP032
8	4	f	occipital	n.a.	II	n.a.	no match	n.a.	ST EP	n.a.	n.a.	n.a.	n.a.	n.a.	Fukuoka et al [5]	EP037
9	2	m	parietal	n.a.	III	n.a.	no match	no fusion	ST EP	n.a.	n.a.	n.a.	n.a.	n.a.	Fukuoka et al [5]	EP092
10	60	f	n.a.	n.a.	II	n.a.	ependymoma, posterior fossa group A	no fusion	ST EP	n.a.	n.a.	n.a.	n.a.	n.a.	Fukuoka et al [5]	EP097
11	3	f	frontal	n.a.	III	n.a.	CNS neuroblastoma with FOXR2 activation	n.a.	ST EP	n.a.	n.a.	n.a.	n.a.	n.a.	Fukuoka et al [5]	EP125
12	22	m	occipital	n.a.	II	n.a.	no match (low score for ependymoma, posterior fossa group B, 0.44)	*FOXO1-STK24*	ST EP with FOXO-STK24 fusion	n.a.	n.a.	n.a.	n.a.	n.a.	Fukuoka et al [5]	EP057
13	45	m	n.a.	n.a.	II	n.a.	no match	no fusion	ST EP with TERT mutation	n.a.	n.a.	n.a.	n.a.	n.a.	Fukuoka et al [5]	EP050
14	7,2	f	supratentorial	n.a.	n.a.	n.a.	n.a.	*C11orf95-YAP1, MYO7A-SESN3*	n.a.	n.a.	n.a.	n.a.	n.a.	n.a.	Parker et al [18]	16
15	2,4	f	supratentorial	n.a.	n.a.	n.a.	n.a.	*C11orf95-MAML2*	n.a.	n.a.	n.a.	n.a.	n.a.	n.a.	Parker et al [18]	17
16	12,8	m	3rd ventricle	*RELA* -like	III	negative	no match (low score for methylation class plexus tumor, 0.54)	no fusion	ST EP, *RELA* -like	HIT-2000 study protocol	7,62	relapse	7,62	dead	this study	1
17	4,2	f	hemispheric	*RELA* -like	III	negative	no match (borderline score for methylation class ependymoma, RELA fusion, 0.89)	no fusion	ST EP, *RELA* -like	HIT-2000 study protocol	11,68	censored	11,68	alive	this study	2
18	1,9	f	hemispheric	*RELA* -like	III	negative	no match	n.a.	ST EP, *RELA* -like	HIT-2000 study protocol	7,68	censored	7,68	alive	this study	3
19	7,2	f	pineal reagion	*RELA* -like	III	negative	no match (low score for methylation class plexus tumor, 0.49)	*C11orf95-NCOA1*	ST EP, *RELA* -like	HIT-2000 study protocol	8,53	censored	8,53	alive	this study	4
20	17,3	m	hemispheric	*RELA* -like	III	negative	ependymoma, RELA fusion	*C11orf95-NCOA1, RTN3-NCOA1*	ST EP, *RELA* -like	HIT-2000/E-HIT 2004 study protocol	7,23	censored	7,23	alive	this study	5
21	0,5	m	lateral ventricle	*RELA* -like	III	negative	no match	no fusion	ST EP, *RELA* -like	VEC-Chemotherapy; UKCCSG/SIOP study	4,9	relapse	5,6	dead	this study	6
22	4,2	f	lateral ventricle	*RELA* -like	III	negative	ependymoma, RELA fusion	n.a.	ST EP, *RELA* -like	HIT-2000 study protocol	5,49	censored	5,49	alive	this study	7
23	6,6	m	hemispheric	*RELA* -like	III	negative	no match	*PLAGL1-EWSR1*	ST EP, *RELA* -like	HIT-2000 study protocol	7,48	censored	7,48	alive	this study	8
24	2,3	m	hemispheric	*RELA* -like	III	negative	no match (low score for methylation class ependymoma, RELA fusion, 0.62)	no fusion	ST EP, *RELA* -like	n.a.	n.a.	n.a.	n.a.	n.a.	this study	9
25	15	m	3rd ventricle	tanycytic	II	negative	no match (low score for methylation class plexus tumor, 0.61)	n.a.	ST EP, tanycytic	HIT-2000 study protocol	10,4	censored	10,4	alive	this study	10
26	13,6	f	hemispheric	tanycytic	III	negative	no match(low score for methylation class subependymoma, posterior fossa, 0.39)	n.a.	ST EP, tanycytic	I-HIT-MED	5,58	censored	5,58	alive	this study	11
27	14,9	m	hemispheric	tanycytic	II	negative	no match	no fusion	ST EP, tanycytic	I-HIT-MED	3,38	censored	3,38	alive	this study	12
28	6,9	f	hemispheric	tanycytic	II	negative	no match (low score for methylation class subependymoma, spinal, 0.32)	no fusion	ST EP, tanycytic	n.a.	n.a.	n.a.	n.a.	n.a.	this study	13
29	17,8	m	hemispheric	tanycytic	II	negative	no match	no fusion	ST EP, tanycytic	Irradiation	2,8	censored	2,8	alive	this study	14
30	10,3	f	pineal reagion	tanycytic	II	negative	no match (low score for methylation class ependymoma, posterior fossa group B, 0.56)	no fusion	ST EP, tanycytic	Irradiation	6,9	censored	6,9	alive	this study	15
31	13,5	f	hemispheric	astroblastoma-like	III	negative	no match (low score for methylation class plexus tumor, 0.48)	*PATZ1-MN1*	ST EP, astroblastoma-like	HIT-2000 study protocol	6,78	censored	6,78	alive	this study	16
32	5,3	f	hemispheric	astroblastoma-like	III	negative	no match	*MYH9-SEC14L2, MTMR3-NCOA3*	ST EP, astroblastoma-like	HIT-2000 study protocol	8,61	censored	8,61	alive	this study	17
33	9,4	m	3rd ventricle	other	III	negative	ependymoma, posterior fossa group B	n.a.	ST EP, PF-B epigenetic profile	I-HIT-MED	1,7	censored	1,7	alive	this study	18

Dx=diagnosis, m=male, f=female, n.a.=not available/applicable, EP=ependymoma, ST=supratentorial, EFS=event-free survival, OS=overall survival

Supplementary Table 1. Summary of characteristics of published cases of non-RELA/non-YAP supratentorial ependymomas and this series.
